# GWAS and Regularised Regression Identify SNPs Associated with Candidate Genes for Stage-Specific Salinity Tolerance in Rice

**DOI:** 10.3390/plants15071046

**Published:** 2026-03-28

**Authors:** Sampathkumar Renukadevi Sruthi, Zishan Ahmad, Anket Sharma, Venkatesan Lokesh, Natarajan Laleeth Kumar, Arulkumar Rinitta Pearlin, Ramanathan Janani, Yesudhas Anbu Selvam, Muthusamy Ramakrishnan

**Affiliations:** 1 Genetics and Plant Breeding, Amrita School of Agricultural Sciences, Amrita Vishwa Vidyapeetham, Coimbatore 642109, India; sruthisampathkumar1998@gmail.com; 2 Department of Genetics and Plant Breeding, Faculty of Agriculture, Annamalai University, Annamalai Nagar, Chidambaram 608002, India; lokiaufoa2018@gmail.com (V.L.); laleethsd12@gmail.com (N.L.K.); rinipearlin@gmail.com (A.R.P.); rm26janani@gmail.com (R.J.); 3 State Key Laboratory of Tree Genetics and Breeding, Co-Innovation Center for Sustainable Forestry in Southern China, Bamboo Research Institute, Key Laboratory of National Forestry and Grassland Administration on Subtropical Forest Biodiversity Conservation, School of Life Sciences, Nanjing Forestry University, Nanjing 210037, China; ahmad.lycos@gmail.com; 4 State Key Laboratory for Development and Utilization of Forest Food Resources, Zhejiang Key Laboratory of Non-Wood Forest Products Quality Regulation and Processing Utilization, Zhejiang A&F University, Hangzhou 311300, China; anketsharma@gmail.com

**Keywords:** rice, salinity, GWAS, SNP, physiology, phenotyping, prediction, plant stress

## Abstract

Soil salinity remains a major constraint to rice productivity, particularly during early developmental stages when plants are highly sensitive to osmotic and ionic stress. In this study, we evaluated 201 genetically diverse rice genotypes from the 3K Rice Diversity Panel to investigate stage-specific mechanisms of salinity tolerance and develop machine learning-based predictive models for rapid phenotypic screening. Morphological and physiological traits were measured under control and saline conditions at germination and early seedling stages to derive Stress Tolerance Indices (STIs). The average membership function value (AMFV), calculated from multi-trait STI profiles, effectively captured variation in salinity responses and enabled classification of genotypes into five tolerance categories. Genome-wide association analysis using high-density SNP markers identified 36 significant marker–trait associations, including potentially novel SNPs on chromosomes 1 and 12. Several loci co-localized with candidate genes (*LTR1*, *LGF1*, *OsCPS4*, *OsNCX7*, and *OsNHX4*), while functional SNPs within genes (*OsDRP2C*, *RLCK168*, and *OsMed37_2*) and non-synonymous variants (*qSVII11.1* and *qSNaK3.1*) further supported their candidacy in salinity tolerance. Mining favourable SNPs of causal genes identified superior multilocus combinations consistent with STI-based phenotypic patterns, with genotype 91-382 emerging as the strongest performer, exhibiting enhanced Na^+^ exclusion, K^+^ retention, and biomass resilience across developmental stages. To address multicollinearity among STI traits, we applied cross-validated LASSO (germination) and Elastic Net (early seedling) models, achieving high predictive accuracy and revealing a developmental shift from biomass-driven tolerance at germination to ion-regulatory processes at the seedling stage. Independent validation showed strong agreement between predicted and observed AMFVs. By integrating physiological indices, GWAS-derived SNP signals, and regularized machine learning approaches, this study provides a robust framework for identifying elite donors and accelerating breeding for salt-tolerant rice.

## 1. Introduction

India faces an intensifying challenge of soil salinization, with approximately 6.74 million hectares already affected and an additional 10% of land becoming salinized each year. If this trend continues, up to 50% of the country’s arable land may be impacted by 2050 [1,2]. Salinity-affected soils occur across four major agriculturally significant ecological regions spanning 15 states and the Andaman and Nicobar Islands. Tamil Nadu falls under both the peninsular and coastal alluvial zones [3], where salinity stress severely limits agricultural productivity. The 2004 tsunami further increased soil salinity along the eastern coast of Tamil Nadu, underscoring the urgent need for sustainable land management and genetic improvement of crops to mitigate its effects.

Rice (*Oryza sativa* L.), the primary food source for half of the global population, is widely cultivated in the eastern coastal regions of Tamil Nadu. Among cereals, rice is the most sensitive to salinity stress [4], with a saturated paste EC threshold of 3 dS/m and yield losses of up to 1 t/ha when floodwater salinity exceeds 2 dS/m [5,6]. This sensitivity is particularly acute during early growth stages [7]. Salinity stress disrupts key physiological processes, including ion homeostasis, osmotic balance, and oxidative regulation, and induces ion toxicity, collectively impairing seed germination, seedling growth, leaf and shoot development, flowering, and overall yield [8,9,10,11,12]. Salt stress is estimated to reduce rice yield by 30–50% annually [13].

Given the severity of salinity stress, especially where land expansion is impractical and agronomic interventions offer limited relief, developing salt-tolerant rice genotypes becomes essential. Advancing this goal requires a clear understanding of salinity tolerance mechanisms and the identification of tolerant genotypes for genetic improvement. Evaluating rice genotypes across multiple developmental stages is crucial [14,15,16], and quantitative stress indices provide robust tools for accurately identifying and differentiating salinity-tolerant genotypes [17]. Among these, the Stress Tolerance Index (STI) is widely used to assess genotypes with high yield potential under both stress and non-stress conditions. Higher STI values indicate stronger tolerance, making it a reliable metric that integrates stability and productivity [18]. Using STI enables breeders to efficiently screen and select genotypes that maintain productivity under saline conditions, thereby accelerating the development of resilient varieties suited to salt-affected regions.

Unlike earlier studies that used STI to assess salinity tolerance [19,20,21], the present work evaluates a larger and genetically richer panel of 201 rice genotypes from the 3000 Rice Genome Project (3K-RGP) [22]. This panel offers exceptional phenotypic and genotypic diversity due to its broad allelic variation and high-resolution SNP coverage, enabling detailed dissection of complex traits. STI values at both germination and early seedling stages reflect phenotypic performance that may be influenced by environmental variation, making genomic validation essential. Because STI is a composite index and cannot be directly used in GWAS, we used individual morphological and physiological traits measured under saline conditions to identify marker–trait associations (MTAs) that biologically support and validate STI-based classification. Integrating dense genomic data with quantitative trait variation enables the discovery of novel SNP loci and favourable alleles governing salinity tolerance, providing strong molecular evidence for the accuracy of STI-based phenotyping.

The expanded sample size not only enhances the identification of genotypes with superior salt tolerance but also supports the development of stage-specific predictive models and the identification of key standalone predictors of salinity tolerance. Importantly, this study is the first to apply machine learning regularization techniques to predict salinity tolerance in rice, establishing a robust and integrated framework for precise selection and genetic improvement of cultivars suited to saline environments.

## 2. Results

The genotypes evaluated for salt tolerance were predominantly from indica-derived groups (indx, ind1A, ind1B, ind2, and ind3), which collectively accounted for more than 80% of the panel. The remaining genotypes belonged to aus, aromatic, japonica, temperate, tropical, and admixture groups (Supplementary Table S1).

### 2.1. STI Differences and Significant Genotypic Responses Under Germination and Early Seedling-Stage Salt Stress

**Figure 1:**
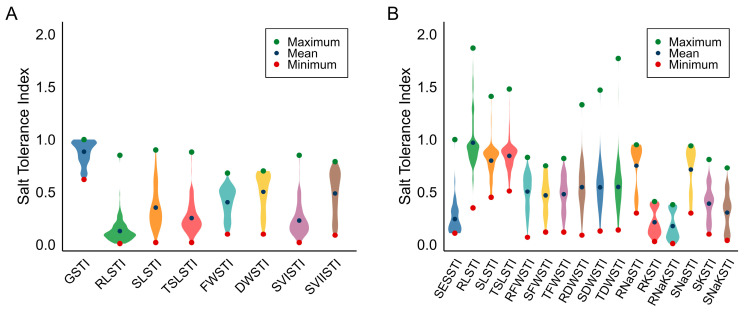
Violin plot illustrating the distribution (mean and range) of STI across 201 rice genotypes in the (**A**) germination stage and (**B**) early seedling stage. Abbreviations in the figure are defined in the List of Abbreviations.

STI was used to assess genotype performance under saline conditions relative to non-stress conditions. STI varied substantially among genotypes at both germination and early seedling stages across all evaluated traits (Figure 1). At the germination stage, variation was observed for STI for germination percentage (GSTI), root length (RLSTI), shoot length (SLSTI), total seedling length (TSLSTI), fresh weight (FWSTI), dry weight (DWSTI), seedling vigour index I (SVISTI), and seedling vigour index II (SVIISTI). Mean STI values were lower for elongation-related traits such as RLSTI, SVISTI, and TSLSTI than for biomass-related traits FWSTI and DWSTI, indicating stronger suppression of axial growth under salinity. Genotype G120 ranked highest across several shoot growth and vigour traits (SLSTI, TSLSTI, SVISTI), whereas G150 consistently recorded the lowest biomass indices. Genotypes G14, G19, G74, G84, G93, and G94 also maintained relatively high STI values across multiple germination traits, suggesting broad-based tolerance at this stage.

At the early seedling stage, greater variation was observed for STI for salt injury score (SESSTI), RLSTI, SLSTI, TSLSTI, root fresh weight (RFWSTI), shoot fresh weight (SFWSTI), total fresh weight (TFWSTI), root dry weight (RDWSTI), shoot dry weight (SDWSTI), total dry weight (TDWSTI), and ion-related traits including root sodium (RNaSTI), root potassium (RKSTI), root Na^+^/K^+^ ratio (RNaKSTI), shoot sodium (SNaSTI), shoot potassium (SKSTI), and shoot Na^+^/K^+^ ratio (SNaKSTI). STI ranges expanded markedly at this stage, with the tolerant check FL478 showing the highest RLSTI (1.87), while G129 exhibited very high TDWSTI (1.77) and SDWSTI (1.47), indicating performance above the non-stressed mean.

Ion-related indices, particularly RKSTI, RNaKSTI, and SNaKSTI, showed lower mean values than growth-related traits, indicating greater sensitivity of ionic balance under salinity. Genotype G84 performed consistently well across both morphological traits (RFWSTI, TFWSTI) and ionic indices (RNaKSTI), whereas G150 remained the weakest genotype across biomass and ionic traits. The differential STI responses across growth, biomass, and ionic traits confirm that salt tolerance is a physiologically complex, trait- and stage-dependent phenomenon with substantial genotypic variation during early plant development.

**Figure 2:**
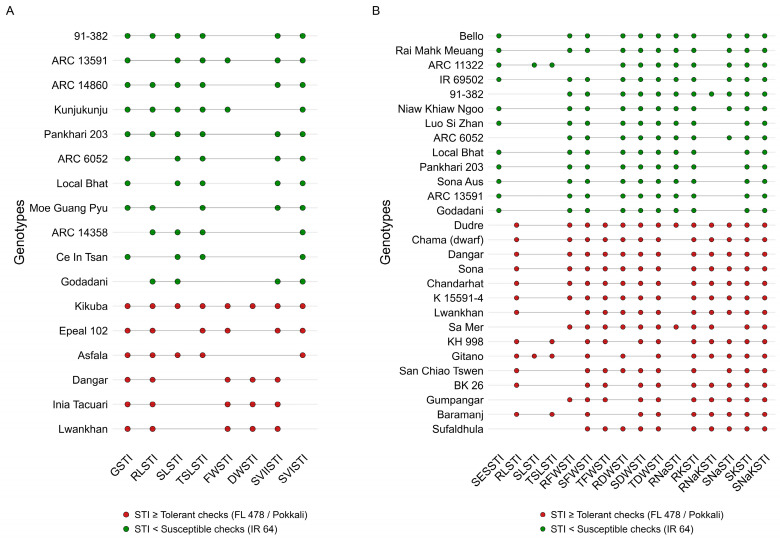
Dot plots highlighting genotypes that outperform tolerant and susceptible checks under salinity stress: (**A**) genotypes exceeding checks in at least four germination-stage STI traits and (**B**) genotypes exceeding checks in at least eight early seedling-stage STI traits. Abbreviations in the figure are defined in the List of Abbreviations.

Figure 2 summarizes the comparative STI responses of genotypes relative to tolerant and susceptible checks at both germination and early seedling stages. A subset of genotypes, namely G48, G70, G76, G84, and G167, consistently exhibited significantly higher STI values than the tolerant check (*p* < 0.05) across the specified number of traits at both stages, indicating stable salinity tolerance. In contrast, G36 and G184 consistently showed significantly lower STI values than the susceptible check (*p* < 0.05) across multiple traits, confirming their stable susceptibility to salinity stress. These consistently contrasting responses identify reliable tolerant and susceptible genotypes that can serve as valuable phenotypic materials for subsequent genetic analysis and salinity tolerance breeding.

### 2.2. Classification of Genotypes Based on AMFV Derived from STI Under Salinity Stress

**Figure 3:**
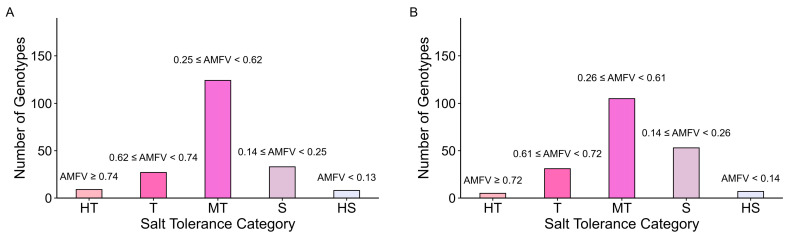
Classification of genotypes based on AMFV under salinity stress: (**A**) germination stage, and (**B**) early seedling stage. AFMV—average membership function value, HT—highly tolerant, T—tolerant, MT—moderately tolerant, S—susceptible, HS—highly susceptible.

MFVs were calculated for each trait based on the corresponding STI, and the average MFV (AMFV) for each genotype was obtained by averaging MFVs across traits. At the germination stage, AMFV ranged from 0.06 (G113) to 0.97 (G120), with a mean of 0.44 ± 0.19, and the 201 genotypes were classified into five salinity tolerance categories based on the AMFV distribution using a standard formula (Table 1; Figure 3; Supplementary Table S2). The highly tolerant genotypes were G22, G48, G51, G53, G70, G76, G84, G120, and G167, whereas the highly susceptible genotypes were G8, G36, G72, G90, G112, G113, G119, and G184. At the early seedling stage, AMFV ranged from 0.08 (G150) to 0.73 (G30, G88), with a mean of 0.43 ± 0.18, and genotypes were similarly grouped into five tolerance classes using the same criteria, with G18, G30, G84, G88, and G94 classified as highly tolerant and G4, G27, G72, G133, G150, G174, and G184 as highly susceptible (Table 1; Figure 3; Supplementary Table S3).

### 2.3. Cross-Stage Dynamics of Salt Tolerance in Rice Genotypes

**Figure 4:**
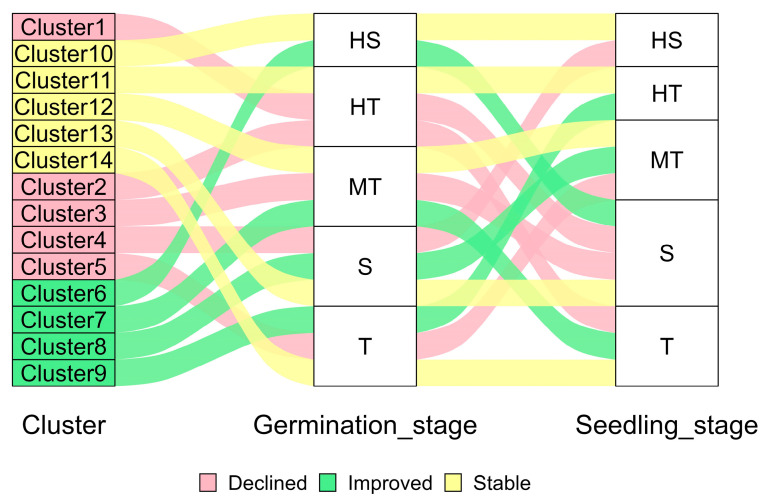
Alluvial plot depicting cross-stage dynamics of salt tolerance in rice genotypes. Flows represent transitions of genotypes from cluster groups at the germination stage to tolerance classes at the seedling stage. Colours indicate response patterns across stages. HT—highly tolerant, T—tolerant, MT—moderately tolerant, S—susceptible, HS—highly susceptible.

Among the 201 rice genotypes analyzed, 27 genotypes (13.4%) improved in salt tolerance classification, 48 genotypes (23.9%) showed a decline, and 126 genotypes (62.7%) remained stable across developmental stages based on AMFVs. Genotypes that improved in tolerance occurred primarily in Clusters 6 to 9 and typically shifted from MT or S categories to T or HT categories. For example, G63 improved from MT at the germination stage to T at the early seedling stage. Genotypes showing a decline in tolerance were distributed across Clusters 1 to 5 and generally shifted from higher tolerance classes (HT or T) to lower ones (MT or S). Stable genotypes, dominant across Clusters 10 to 14, included 96 genotypes in Cluster 12 and were most frequently classified within the MT category at both stages. Notably, G84 (Cluster 11) consistently maintained an HT classification across stages, demonstrating stable salt tolerance, whereas G51 and G76 (Cluster 10) remained in the HS category, indicating persistent susceptibility (Figure 4). Complete classification details appear in Supplementary Table S4.

### 2.4. Variation Inflation Factor and Multicollinearity Among Stress Tolerance Indices

We divided the 201 rice genotypes into training and testing populations using stratified sampling across the germination and early seedling stages. VIF analysis of the training sets at both stages revealed substantial multicollinearity, with most predictor traits showing high VIF values (>10), indicating considerable multicollinearity.

### 2.5. Predictive Modelling of Rice Salt Tolerance Using LASSO and Elastic Net Regularization

We developed predictive models for salt tolerance at the germination and early seedling stages using regularized regression, based on training sets of 160 and 159 genotypes, respectively, to effectively handle multicollinearity among STI traits. During model tuning, we applied k-fold cross-validation to identify the optimal α value by selecting the model with the lowest MSE and highest R^2^. The resulting α determined whether the final model behaved as LASSO (α = 1), Ridge (α = 0), or Elastic Net (0 < α < 1).

At the germination stage, cross-validation identified an optimal α value of 1, which produced the lowest MSE and highest R^2^, indicating that a LASSO regression model provided the best predictive performance (Supplementary Table S7). The resulting model generated a sparse solution with an intercept of −0.220 and seven significant predictors: GSTI (β = 0.282), RLSTI (β = 0.097), SLSTI (β = 0.108), FWSTI (β = 0.219), DWSTI (β = 0.197), SVISTI (β = 0.373), and SVIISTI (β = 0.173). TSLSTI was excluded due to its zero coefficient. The model showed an excellent fit, explaining 99.9% of the variance (R^2^ = 0.9990; Adjusted R^2^ = 0.9990), with minimal error (MSE = 3.19 × 10^−5^, RMSE = 0.0057, NMSE = 0.0008). The optimal regularization parameter was λ = 0.0027 (λ_min), while a slightly higher λ = 0.0032 (1 SE rule) produced a simpler model with negligible loss of accuracy (Supplementary Table S7).

**Figure 5:**
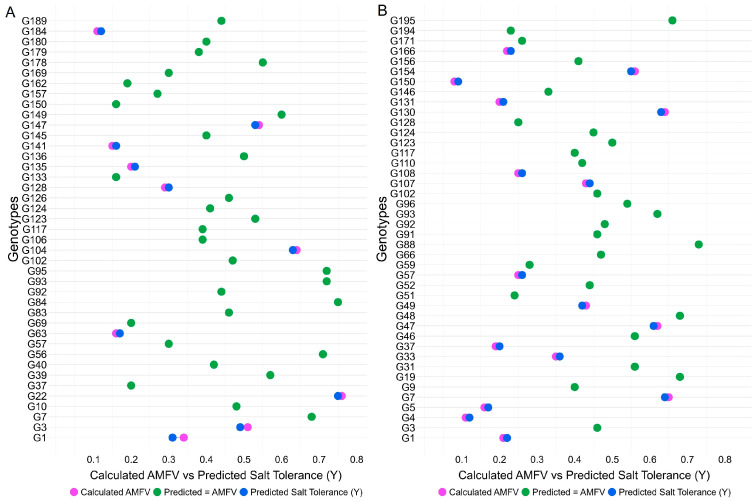
Comparison of predicted salt tolerance (Y) and calculated AMFV in the testing population: (**A**) germination stage, and (**B**) early seedling stage; AMFV—average membership function value.

The regression equation was: Y = −0.220 + 0.282 × GSTI + 0.097 × RLSTI + 0.108 × SLSTI + 0.219 × FWSTI + 0.197 × DWSTI + 0.373 × SVISTI + 0.173 × SVIISTI. Validation using 41 test genotypes showed strong agreement between predicted values and experimentally derived MFVs. Ten genotypes showed very close matches (e.g., G3: MFV = 0.49 vs. Y = 0.51; G22: MFV = 0.75 vs. Y = 0.76), and 31 genotypes showed identical values (e.g., G7: MFV = 0.68 vs. Y = 0.68) (Figure 5). At the early seedling stage, cross-validation identified an optimal α value of 0.1, which produced the lowest MSE and highest R^2^, indicating that Elastic Net regularization provided the best predictive performance. The final model retained all STI predictors with an intercept of −0.183, with RNaKSTI showing the highest coefficient (0.162), followed by RKSTI (0.151) and SESSTI (0.070), while RLSTI contributed the least (0.028). The model also demonstrated an excellent fit (Supplementary Table S8).

The regression equation was: Y = −0.183 + 0.070 × SESSTI + 0.028 × RLSTI + 0.042 × SLSTI + 0.089 × TSLSTI + 0.099 × RFWSTI + 0.082 × SFWSTI + 0.090 × TFWSTI + 0.058 × RDWSTI + 0.048 × SDWSTI + 0.040 × TDWSTI + 0.081 × RNaSTI + 0.151 × RKSTI + 0.162 × RNaKSTI + 0.093 × SNaSTI + 0.084 × SKSTI + 0.097 × SNaKSTI. Validation with 42 test genotypes again showed high predictive accuracy, with 15 genotypes showing nearly identical values and 26 genotypes matching exactly (Figure 5).

### 2.6. Identification of Key Predictors of Salt Tolerance

**Figure 6:**
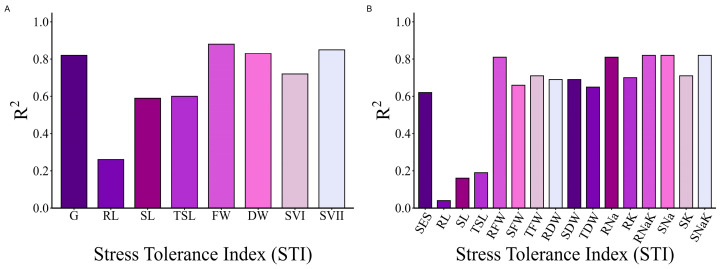
Coefficient of determination (R^2^) between MFV and STI of individual traits under salinity stress: (**A**) germination stage and (**B**) early seedling stage. Abbreviations in the figure are defined in the List of Abbreviations.

To identify key predictors of salt tolerance, we calculated the coefficient of determination (R^2^) between MFV and the STI of individual traits at both developmental stages using the training population. At the germination stage, FWSTI showed the strongest association (R^2^ = 0.88), followed by SVIISTI (0.85), while RLSTI showed the weakest (0.26) (Figure 6). At the early seedling stage, SNaKSTI, RNaKSTI, and SNaSTI each recorded the highest R^2^ values (0.82), whereas RLSTI again showed the weakest association (R^2^ = 0.04) (Figure 6). Comparable trends in the testing population confirmed the robustness and reproducibility of these relationships across independent datasets.

### 2.7. Genome-Wide Association Mapping

**Figure 7:**
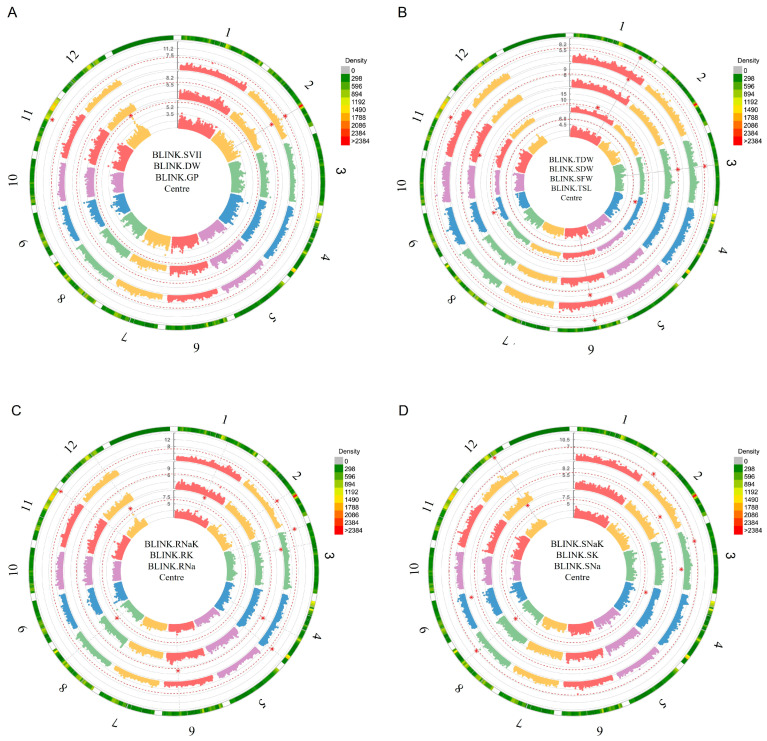
Circular Manhattan plots depicting significant MTAs for salinity tolerance in rice under saline conditions. Chromosomes are arranged sequentially around the circumference, and concentric circles represent individual traits, with the first trait listed corresponding to the innermost circle and subsequent traits arranged outward. Each point denotes an SNP plotted by genomic position, with radial distance indicating −log_10_ (*p*-value). The horizontal line represents the genome-wide significance threshold, and red star symbols indicate peak SNPs (major MTAs). Numerals correspond to chromosome numbers, and each color represents a different chromosome. (**A**) Germination stage—GP, DW, SVII; (**B**) early seedling stage—TSL, SFW, SDW, TDW; (**C**) early seedling stage—RNa, RK, RNaK; (**D**) early seedling stage—SNa, SK, SNaK. Abbreviations are defined in the List of Abbreviations.

GWAS identified 36 SNPs significantly associated with salinity tolerance traits under saline conditions (Supplementary Table S5). Circular Manhattan and Q–Q plots illustrating representative associations are shown in Figure 7 and Supplementary Figure S1. At the germination stage (Figure 7A), Manhattan peaks were concentrated on two chromosomes. On chromosome 2, qDW2.1 explained 52.56% of phenotypic variation with an 83.33% allelic advantage, while qSVII11.1 on chromosome 11 showed a higher 97.78% allelic advantage with 37.99% PVE. This narrow chromosomal distribution indicates that germination-stage salt tolerance is largely controlled by a few loci with strong effects on early biomass accumulation and seedling vigour.

At the early seedling stage (Figure 7B–D), association peaks were distributed across nine chromosomes (1, 2, 3, 4, 6, 8, 9, 11, and 12), indicating a broader genetic basis. The strongest signal corresponded to qSFW11.1 on chromosome 11 (178.92% allelic advantage, 54% PVE). Major ionic loci included qRK3.1 (193.02%, 51.56% PVE), qRNa12.1 (61.31%, 42.26% PVE), qRNaK11.1 (61.76%, 39.83% PVE), and qSNa12.1 (62.70%, 38.13% PVE). Potassium-related loci qSK9.1, qSK3.1, and qSNaK12.1 also showed >60% allelic advantage and >34% PVE, highlighting genomic control of potassium retention and Na^+^/K^+^ balance.

Several SNPs were clustered in the same genomic regions, including qDW2.1–qSVII2.1, qTSL4.1–qSNa4.1, qSDW2.1–qTDW2.1, qSDW3.1–qTDW3.1, qSDW6.1–qTDW6.1, as well as qGP12.1, qRNa12.1, qSNa12.1, and qSNaK12.1, indicating co-association across traits and pleiotrophy within and across stages (Supplementary Figure S2). Examination of genomic regions surrounding significant SNPs (±150 kb) showed that the identified peak SNPs of this study were located within and near annotated rice genes (Supplementary Table S5 and Table 2). 

Notably, SNPs associated with qSFW1.1 (chromosome 1) and qGP12.1, qGP12.2, qRNa12.1, qSNa12.1, and qSNaK12.1 (chromosome 12) were detected as potentially novel loci. Several loci were located near candidate genes, including ASMT1, MDH1, OsPP2C8, OsPHD9, OsBADH2, OsRSZ23, OsSEC3A, and OsFes1C. Additionally, SNPs associated with qRNa8.1, qRNaK4.1, and qSNaK2.1 were located within the genes OsDRP2C, RLCK168, and OsMed37_2, respectively. Non-synonymous SNPs were identified at qSVII11.1 and qSNaK3.1 (Table 2).

**Figure 8:**
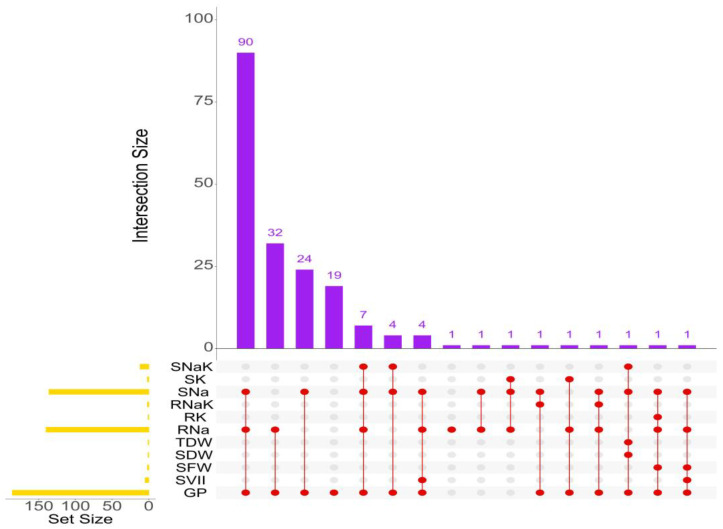
Upset plot showing genotype groups with superior allelic combinations across traits. Yellow bars indicate the number of selected genotypes per trait; connected red dots indicate overlap among traits. Traits: GP, SVII (germination stage); SFW, SDW, TDW, RNa, RK, RNa/K, SNa, SK, and SNa/K (early seedling stage). Abbreviations in the figure are defined in the List of Abbreviations.

Favourable and alternate alleles were identified for each of the 36 MTAs based on phenotypic effects (Supplementary Table S5). For example, at qGP12.1, allele T showed a higher germination percentage (83.63%) than allele A (67.17%), providing an allelic advantage of 25.50%. Homozygous allelic combinations were analyzed for each trait with more than one peak SNP (Table 3 and Supplementary Table S6). For example, four allelic combinations, viz., TT, CT, TC, and CC, were identified for SVII. The combination TT produced the highest mean value (1.19), whereas CC produced the lowest (0.49). Across traits, we also examined superior allelic combinations to identify genotypes consistently carrying such favourable SNP patterns. This analysis highlighted genotypes with advantageous combinations at both developmental stages (Figure 8). G47, G84, and G103 carried favourable allelic combinations for up to five traits.

## 3. Discussion

Salt tolerance remains a major constraint in rice, a typical glycophyte, because salinity reduces production in coastal regions worldwide [23]. High salinity disrupts key metabolic processes, impairs germination and seedling establishment, and ultimately reduces plant growth and grain yield. Genotype-specific responses and stage-dependent sensitivity complicate reliable evaluation of salinity tolerance [24]. Here, we integrate phenotypic screening at germination and early seedling stages with multi-trait classification, GWAS, and predictive modelling to identify robust donors and loci for breeding.

### 3.1. Genetic Diversity of the Panel Supports Robust Inference

We evaluated a genetically diverse panel representing major rice subpopulations. Although indica-derived groups predominated, we included aus, aromatic, japonica (temperate and tropical), and admixed accessions, ensuring broad representation. This structure strengthens inference on phenotypic and genetic variation and aligns with established indica–japonica classifications [25,26]. The large sample size also increased statistical power and improved the precision of STI-based tolerance estimates.

### 3.2. STI Captures Germination-Stage Variation and Identifies Early Growth Sensitivity

Previous studies support the Salt Tolerance Index (STI) as a practical tool for salinity screening in rice [27]. However, studies disagree on germination-stage tolerance, reporting both relative tolerance [28,29] and susceptibility [30,31]. This disagreement likely underscores the need for a standardised, multi-trait STI framework rather than reliance on single-trait thresholds. We therefore used STI to compare genotypes consistently at the germination stage. STI varied across eight germination-related traits, underscoring the complexity of early-stage salt responses.

GP showed the highest mean STI, whereas RL showed the lowest, indicating that salinity constrains early seedling growth more strongly than germination itself. Roots encounter salt first; seedlings experience osmotic stress initially, followed by ionic toxicity as Na^+^ and Cl^−^ accumulate. Ion accumulation disrupts cytosolic homeostasis and restricts uptake of essential nutrients (Ca^2+^, Mg^2+^, Fe^2+^, Zn^2+^), which suppresses root expansion and weakens early vigour [32,33,34,35,36,37,38]. Genotype performance mirrored these trait patterns. Genotypes viz., ARC 14860, 91-382, Pankhari 203, ARC 13591, Kunjukunju, ARC 6052, Local Bhat, Moe Gaung Pyu, ARC 14358, Ce In Tsan, and Godadani maintained high STI values for germination traits, suggesting coordinated mechanisms that support germination and early vigour under salinity. In contrast, Kikuba, Epeal 102, Dangar, Inia Tacuari, Lwankhan, and Asfala consistently showed low STI values, indicating stronger osmotic and ionic constraints typical of salt-sensitive lines [21,27,29,39,40,41]. Earlier work likewise showed that STI differentiates genotypes based on germination, biomass, vigour, and Na^+^/K^+^ traits [20,42,43,44,45].

### 3.3. Early Seedling Screening Reveals Tolerance Beyond Visual Scoring

While germination offers an initial tolerance signal, early seedling growth provides a more sensitive and physiologically meaningful test of whether tolerance is sustained under saline conditions. Rice is particularly vulnerable during the first one to three weeks after emergence, and genotypic variation at this stage is well documented [46]. Given the practical constraints of reproductive-stage screening, early seedling assays under controlled conditions remain the preferred approach for rapid, scalable germplasm evaluation [47,48,49,50].

In our panel, SESSTI provided a useful first-pass visual estimate. Genotypes Bello and IRRI 146 (SESSTI = 1) showed no chlorosis, necrosis, or loss of vigour, consistent with established scoring criteria [51]. However, STI-based trait profiling revealed physiological variation that visual scores failed to capture, with several genotypes outperforming visually tolerant lines across growth, biomass, and ionic dimensions. Notably, 91-384 maintained high STI values for Na^+^ exclusion, K^+^ retention, and Na^+^/K^+^ ratio in both roots and shoots, indicative of efficient ionic homeostasis and selective ion transport mechanisms widely recognized as central to salinity tolerance in rice [52,53]. Conversely, several genotypes, including G63, G30, G121, G172, G2, G8, G129, and G85 alongside FL478, recorded STI values exceeding unity for seedling length and biomass, suggesting growth maintenance or even stimulation under salinity. These responses likely reflect osmotic adjustment via compatible solutes, hormonal regulation through gibberellin–cytokinin signalling, Na^+^ sequestration mediated by transporters such as SOS1 and NHX1, and strengthened antioxidant defence systems [54,55,56,57]. In contrast, low STI genotypes exhibited marked growth suppression and elevated Na^+^/K^+^ ratios, indicating disrupted ionic balance and reduced stress adaptation. Critically, the failure of visual scoring to identify these physiological distinctions argues against its use as a primary screening tool in germplasm evaluation programmes where mechanistic differentiation is required.

These findings support integrated hydroponic screening frameworks that combine morphological, physiological, and ionic traits, and further confirm the effectiveness of STI in reliably identifying salt-tolerant rice genotypes based on root and shoot growth and biomass responses at the seedling stage [15,58,59]. Collectively, the consistent high-STI performance of IR 69502-6-SRN-3-UBN-1-B, Bello, ARC 6052, ARC 11322, Sona Aus, ARC 13591, Local Bhat, Godadani, 91-382, Niaw Khiaw Ngoo, Rai Mahk Meuang, Luo Si Zhan, and Pankhari 203 across trait classes reflects coordinated, multi-mechanistic tolerance and affirms these genotypes as priority candidates for salt-tolerant rice breeding.

### 3.4. AMFV Consolidates Multi-Trait Tolerance and Strengthens Classification

AMFV integrates STIs across multiple traits, enabling genotypes to be classified based on overall physiological performance rather than a single trait. This is important because individual trait indices are inevitably influenced by developmental variation and environment-specific noise; averaging across traits reduces this variance and yields a more stable tolerance estimate. Compared with single-trait STI approaches, this multi-trait framework provides a more robust assessment of salinity tolerance because individual traits often capture only specific aspects of the stress response and may vary across environments. By combining growth, biomass, and ionic regulation traits, AMFV reflects coordinated physiological mechanisms underlying salt tolerance and reduces the risk of misclassification associated with single-trait selection.

In this study, most genotypes maintained consistent tolerance classes from germination to early seedling stages, indicating that the physiological basis of tolerance in these genotypes is not stage-restricted but reflects stable intrinsic capacity across early development. Notably, 91-382 was consistently classified as highly tolerant, consistent with its strong Na^+^ exclusion, K^+^ retention, Na^+^/K^+^ balance, and biomass stability, whereas K 15591-4 and Dangar were consistently classified as highly susceptible, indicating sustained physiological instability under salinity. Similar advantages of MFV-based multi-trait classification have been reported previously [42,43,60]. However, because AMFV averages trait responses, it may reduce sensitivity to specific tolerance mechanisms and obscure the contribution of individual traits with strong biological significance. Despite this limitation, AMFV provides a practical framework for identifying stable donor genotypes, and integrating this phenotypic classification with genome-wide association studies (GWAS) can help identify SNPs and favourable alleles underlying salinity tolerance.

### 3.5. Regularised Regression Improves Prediction Under Multicollinearity

Because stress-response traits co-vary strongly, multicollinearity can distort conventional regression. Regularised regression addressed this problem by stabilising coefficient estimates and improving prediction. LASSO (L1) selected informative predictors by shrinking redundant coefficients to zero, whereas Elastic Net combined L1 and L2 penalties to manage correlated predictors.

Cross-validation selected LASSO for germination and Elastic Net for the early seedling stage, indicating stronger trait interdependence later in development. In the germination-stage model, GSTI, FWSTI, and SVISTI drove prediction, whereas TSLSTI added no value once root and shoot lengths entered the model. In the early seedling model, Elastic Net retained all predictors and emphasised ion-regulatory traits (notably RNaKSTI and RKSTI), highlighting the importance of root Na^+^/K^+^ balance. The high predictive performance and close agreement between predicted values and observed AMFVs validate these models as practical tools for screening large panels where measuring all traits is resource-prohibitive, and support their deployment as phenotypic pre-selection filters in breeding pipelines.

### 3.6. Single-Trait Regression Supports Stage-Specific Mechanisms

Univariate regression revealed a clear developmental shift in trait importance. At germination, FWSTI and SVIISTI explained the greatest variation, emphasizing the role of early biomass and vigour under osmotic stress, whereas RLSTI contributed little explanatory power [42,60]. By the early seedling stage, ionic indices (SNaKSTI, RNaKSTI, SNaSTI) became the dominant predictors, indicating that tolerance increasingly depends on ion regulation rather than root elongation. This transition suggests that biomass maintenance governs early performance, while ionic homeostasis becomes critical as ionic toxicity develops, which may explain why studies lacking ion-based indices emphasized fresh weight traits [20]. Such stage-dependent responses reflect the typical monocot salinity strategy, where tolerance relies on maintaining Na^+^ exclusion and favourable Na^+^/K^+^ ratios via HKT-mediated Na^+^ retrieval and NHX-mediated vacuolar sequestration [61,62]. In contrast, dicots such as Arabidopsis and blackgram often depend more on root-based SOS signalling and root architectural adjustments to mitigate salt stress [63,64]. Accordingly, the consistently low contribution of RLSTI indicates that root length reduction in rice reflects stress injury rather than an adaptive tolerance trait, limiting its usefulness as a primary selection criterion in monocot salinity screening.

### 3.7. GWAS Identifies Novel and Gene-Supported Loci Across Stages

**Figure 9:**
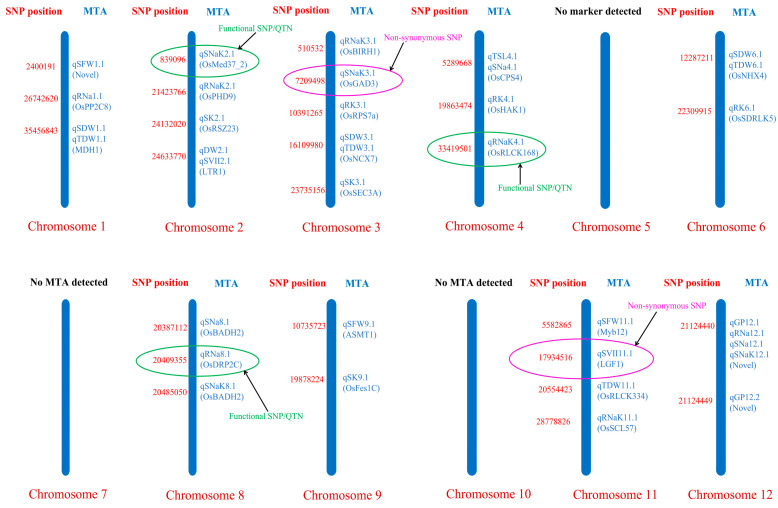
Genome-wide distribution of significant marker–trait associations (MTAs) for salinity tolerance-related traits across the 12 rice chromosomes. Each vertical blue bar represents a chromosome (Chr 1–12). SNP positions are shown in red on the left of each chromosome, and the corresponding associated traits/QTLs are shown in blue on the right. Putative candidate genes underlying major MTAs are indicated in parentheses. Green circles denote functional SNPs (SNPs located within candidate genes), while pink circles denote non-synonymous SNPs. SNPs without proximal candidate genes were regarded as novel.

Genome-wide association analysis (GWAS) was conducted to refine genomic regions associated with salinity tolerance and to identify candidate genes underlying trait variation across germination and early seedling stages. Compared with traditional linkage-based QTL mapping, GWAS exploits historical recombination within diverse germplasm panels and therefore provides higher mapping resolution for detecting functional loci. The analysis confirmed the genetic basis of salinity tolerance and revealed several novel functional, and pleiotropic loci operating across germination and early seedling development (Figure 9). 

A clear developmental shift in genetic architecture was evident: tolerance during germination is driven by a few large-effect loci, whereas early seedling tolerance involves multiple loci distributed across the genome, reflecting a transition from simple to polygenic genetic control during early growth under salinity stress. We detected significant SNPs (qSFW1.1, qGP12.1, qGP12.2, qRNa12.1, qSNa12.1, qSNa/K12.1) located in genomic regions where no salinity-related loci have previously been reported, suggesting the discovery of potentially novel genomic regions contributing to salt tolerance in rice [65].

Several detected loci co-localized with established salt-response genes, indicating that our SNPs likely affect these well-characterized pathways. At germination, when osmotic stress dominates, qDW2.1 and qSVII2.1 mapped near LTR1, a wax-synthesis gene whose loss-of-function increases salt sensitivity by disrupting water and ion homeostasis [66]. Similarly, qSVII11.1 localized between LGF1 and a GRAS transcription factor [67]—both implicated in root barrier formation and seedling vigour regulation—functions critical during osmotic stress. At the early seedling stage, when ionic toxicity becomes limiting, associated loci were predominantly linked to genes regulating metabolic adjustment, ion homeostasis, and stress signalling. The locus qTSL4.1 corresponded to OsCPS4, which participates in diterpenoid biosynthesis, including gibberellins and phytoalexins that contribute to stress tolerance [68,69,70,71]. Antioxidative protection and growth regulation were associated with qSFW9.1, linked to ASMT1, a melatonin biosynthesis enzyme whose suppression reduces melatonin levels and increases salinity sensitivity [72], while qSFW11.1 corresponded to MYB12, a transcription factor regulating abiotic stress tolerance. Metabolic homeostasis was represented by qSDW1.1 associated with MDH1, which modulates vitamin B6 homeostasis and salt sensitivity [73].

Loci qSDW3.1 and qSDW6.1 corresponded to OsNCX7 and OsNHX4, regulators of cytosolic Ca^2+^ signalling and vacuolar Na^+^/K^+^ sequestration essential for maintaining ionic balance under salinity stress [74,75]. Stress signalling pathways were associated with qRNa1.1 linked to OsPP2C8, qRK3.1 associated with OsRPS7a, and qRNaK2.1 corresponding to OsPHD9, which regulates stress-responsive genes controlling Na^+^/K^+^ homeostasis [76,77,78]. Osmotic regulation and stress adaptation were further represented by qSNa8.1 linked to OsBADH2 and qSK9.1 corresponding to OsFes1C, genes implicated in salt stress responses and cellular protection [79,80]. The diversity of gene-associated loci indicates that salinity tolerance in this panel is governed by SNPs involved in multiple salt-response mechanisms, highlighting promising genomic targets for marker-assisted selection and allele pyramiding in rice.

### 3.8. Functional SNPs and Pleiotropy Nominate Breeding Targets

Within-gene SNPs represent strong candidates for gene discovery because sequence variants within coding regions are more likely to influence gene function than variants located in distant genomic regions. We identified several such functional SNPs: qRNa8.1 mapped within OsDRP2C, implicating membrane trafficking and ion-transport regulation [81]; qRNa/K4.1 within RLCK168, linking detected variation to antioxidant defences and ABA signalling [82]; qSNaK2.1 within Os-Med37_2, anchoring SNPs to mediator-driven transcriptional control of ion homeostasis [83]. This SNP-to-gene resolution, which identifies specific nucleotides within candidate genes rather than reporting genomic intervals, marks the transition from QTL mapping to candidate gene identification.

Non-synonymous SNPs (qSVII11.1, qSNaK3.1) further strengthen candidate status: amino acid substitutions directly alter protein structure and function, making these variants mechanistically plausible causal alleles. Critically, multi-trait pleiotropy across developmental stages validates that identified SNPs affect core tolerance mechanisms, not spurious associations. Pleiotropic qGP12.1 co-associated with ion accumulation (qRNa12.1, qSNa12.1), ion balance (qSNaK12.1), and early vigour across both germination and seedling stages, a pattern unlikely by chance and indicating that this locus harbours genes controlling coordinated physiological responses to salt stress. Broad chromosomal distribution confirms that tolerance arises from polygenic architecture, not from major-effect loci. Together, the presence of within-gene SNPs, non-synonymous variants, and stage-spanning pleiotropic loci strengthens their candidacy as key determinants of salinity tolerance, with the identified significant SNPs offering promising targets for functional marker development, marker-assisted selection, and allele pyramiding in rice breeding programmes.

Recent advances in multi-omics integration, combining phenomics, transcriptomics, and metabolomics, are reshaping the discovery of salinity tolerance mechanisms by linking physiological traits with their underlying molecular networks. Approaches such as metabolome-GWAS have demonstrated how metabolic traits can be directly associated with genomic loci controlling processes such as polyamine metabolism, oxidative regulation, and ion homeostasis [84]. Although the present study employed trait-based GWAS using physiological and ionic STIs, several identified candidate genes correspond to pathways frequently highlighted in multi-omics studies. In particular, ASMT1, BADH2, and MDH1 are involved in metabolic and osmotic regulation, whereas OsNHX4 contributes to ionic homeostasis, and OsPP2C8 and OsPHD9 participate in stress signalling and transcriptional regulation. The convergence between physiological trait variation and biologically coherent candidate genes therefore strengthens confidence in the genomic architecture underlying salinity tolerance identified here. Integrating the multi-trait phenotyping and GWAS framework established in this study with future transcriptomic and metabolomic validation will help clarify gene function and facilitate marker development, allele pyramiding, and genomic-assisted breeding for salt-tolerant rice varieties.

### 3.9. Favourable Alleles Validate Phenotypes and Nominate Donor Genotypes

Multilocus profiling identified 91-382, ARC 13502, and Gao Jiao Ying Gan Zhan as leading candidates, each carrying five favourable alleles. However, 91-382 carried a stronger suite of favourable allelic combinations for germination, vigour, biomass, and sodium regulation, giving it the most enriched profile. STI rankings independently identified 91-382 as superior across stages, and GWAS corroborated this assessment, providing a three-way convergence of phenotypic, multi-trait classification, and genomic evidence that is rarely achieved in a single study and substantially elevates confidence in its value as a donor for allele pyramiding and cultivar development.

## 4. Materials and Methods

### 4.1. Plant Materials

A panel of 251 rice accessions originating from 37 countries was assembled from the 3K Rice Genome Diversity Panel (3K-RDP), sourced through the Indian Institute of Rice Research (IIRR), Hyderabad, and the International Rice Research Institute (IRRI), Philippines. From this panel, 201 genotypes representing eight subpopulation groups were evaluated for salinity tolerance at the germination and early seedling stages. The experimental set included the salt-tolerant checks Pokkali and FL478 and the salt-susceptible check IR64. Details on genotype code, genotype name, IRGC accession number, and population type are provided in Supplementary Table S1.

### 4.2. Germination-Stage Screening (Petri Plate Assay)

Germination-stage screening was conducted under controlled conditions in the Seed Technology Laboratory, Department of Genetics and Plant Breeding, Annamalai University, using a Petri plate assay. The experiment followed a completely randomised design (CRD) with three replications per genotype under two treatments (control and salinity stress), with 10 seeds per replication. Uniform, healthy seeds were surface-sterilised with 0.1% mercuric chloride for 5 min, rinsed thoroughly with distilled water, and briefly air-dried to avoid diluting treatment solutions. Sterile 90 mm Petri plates were lined with two layers of germination paper, and 10 seeds were placed evenly in each plate. Each plate received 5 mL of solution: distilled water for controls and 120 mM NaCl for salinity treatment. This concentration lies within the commonly used 50–120 mM range for distinguishing tolerant and sensitive rice genotypes at germination and early seedling stages [4,9,46,85], and concentrations above 100 mM inhibit protein synthesis in salt-sensitive glycophytes [86], providing an effective but non-lethal stress level for screening.

Plates were covered and incubated at 25 ± 2 °C under a 12 h light/12 h dark photoperiod, with relative humidity maintained at 70–75%. Moisture levels were monitored daily and replenished as required according to treatment. Salinity stress was imposed from day 1 and maintained throughout the experiment. Measurements were recorded separately for control and salt-stressed sets on day 8. The following traits were recorded: germination percentage (GP, per cent), root length (RL, cm), shoot length (SL, cm), total seedling length (TSL, cm), fresh weight (FW, g), and dry weight (DW, g) (after oven-drying to constant weight). Seedling vigour index I (SVI) was calculated as GP × TSL, and seedling vigour index II (SVII) was calculated as GP × DW.

### 4.3. Early Seedling-Stage Screening (Hydroponics)

Early seedling-stage screening was conducted under hydroponic conditions in a controlled glasshouse at the Department of Genetics and Plant Breeding, Annamalai University, maintained at 30 °C (day), 20 °C (night), approximately 70% relative humidity, and a 16 h photoperiod. Seeds were surface-sterilised as described above and germinated using the roll-towel method. Five-day-old seedlings were transplanted into plastic trays (41 × 28 × 14 cm) containing 10 L of modified Yoshida nutrient solution [87]. A styrofoam mesh (34 × 24 cm) supported seedlings, with the solution maintained approximately 1 mm above the mesh. Radicles were gently inserted through the mesh to ensure anchorage. The experiment followed a CRD with three replications, with 10 seedlings per genotype per replication. Three days after transplanting, salinity stress was imposed by adding NaCl to reach EC 6 dS m^−1^. After three additional days, EC was increased to 12 dS m^−1^, equivalent to approximately 120 mM NaCl. The nutrient solution was replaced every 7 days, and pH was adjusted daily to 5.0. A non-salinised control (EC 1.4 dS m^−1^) was maintained under identical conditions without NaCl.

Visual scoring of salinity injury was performed 12 days after initial salinisation using the modified IRRI Standard Evaluation System (SES) [51]. SES scoring was omitted in controls because no stress symptoms were observed. At harvest, ten plants per genotype were selected for morphological, biomass, and ionic measurements. Sixteen traits were recorded: SES, RLs (cm), SLs (cm), TSLs (cm), RFWs (g), SFWs (g), TFWs (g), RDWs (g), SDWs (g), TDWs (g), RNa (mmol g^−1^ DW), RK (mmol g^−1^ DW), RNaK, SNa (mmol g^−1^ DW), SK (mmol g^−1^ DW), and SNaK. RLs and SLs were measured in centimeters using a ruler, from the root tip to the base of the shoot (for RL) and from the base to the shoot apex (for SL). Total seedling length (TSL) was calculated as the sum of RL and SL. For dry weight determination, samples were oven-dried at 80 °C for 72 h and weighed using a high-precision digital balance.

### 4.4. Na^+^ and K^+^ Estimation and Salinity Tolerance Index (STI) 

Na^+^ and K^+^ concentrations in root and shoot tissues were estimated using acid digestion followed by flame photometry. Powdered samples were digested in a 9:2 mixture of nitric acid and perchloric acid, diluted to 10 mL with distilled water, and analysed using a Systronics Type 128 flame photometer. Ion concentration (mmol g^−1^ DW) was calculated by multiplying the flame photometer reading (mg L^−1^) by the final digest volume (0.01 L) and dividing by the product of sample dry weight and molar mass (23 g mol^−1^ for Na; 39.1 g mol^−1^ for K). Salinity response was quantified using the STI for each trait, following [60]: STI = Trait value under control/Trait value under stress. STI expresses proportional performance under non-saline relative to saline conditions and enables comparison across traits and genotypes. STI is dimensionless.

### 4.5. Statistical Analysis and Visualisation

All downstream analyses were performed in R (v4.4.1) using STI values from both stages. Descriptive statistics (mean, minimum, maximum, standard error) were calculated, and violin plots were generated to visualise trait distributions across genotypes. Genotypes with STI values significantly higher than the tolerant checks (*p* < 0.05) and significantly lower than the susceptible checks (*p* < 0.05) were identified and visualised using dot plots.

### 4.6. MFV and AMFV-Based Classification

The membership function value (MFV) (0–1) was used to scale genotype performance for each trait, with higher values indicating superior performance. MFVs were calculated from STI as: MFV = X − $X_{min}$/$X_{max}$ − $X_{min}$; where X is the genotype STI for a trait and $X_{min}$ and $X_{max}$ are the minimum and maximum STI values for that trait across genotypes. The average membership function value (AMFV) was calculated for each genotype by averaging MFVs across traits, providing a single multi-trait tolerance score. Following [88], genotypes were classified into five tolerance groups using the AMFV mean ($\bar{X}$) and standard deviation (SD): highly tolerant (HT), tolerant (T), moderately tolerant (MT), susceptible (S), and highly susceptible (HS), using the thresholds: HT if AMFV ≥ $\bar{X}+1.64$SD; T if $\bar{X}+1.64$SD > AMFV ≥ $\bar{X}+1$SD; MT if $\bar{X}+1$SD > AMFV ≥ $\bar{X}-1$SD; S if $\bar{X}-1$SD > AMFV ≥ $\bar{X}-1.64$SD; and HS if AMFV < $\bar{X}-1.64$SD.

### 4.7. SNP Dataset and Quality Control

SNP genotype data for the 3K Rice Diversity Panel (3K-RDP) were obtained from the Rice SNP-Seek database (https://snp-seek.irri.org/, accessed on 14 March 2026), which hosts genomic variation data generated by the 3K Rice Genome Project. The project sequenced 3024 rice accessions and identified SNPs through whole-genome resequencing and variant-calling pipelines, with raw sequence and variant data publicly available through repositories such as AWS S3, GigaDB, and the NCBI Sequence Read Archive (PRJEB6180) [22,89,90]. From this resource, 404,388 SNP markers for 201 genotypes were retrieved and used for GWAS. Quality control was performed in TASSEL v5.2.94 [91]. Genotypes with >20% missing SNP data, markers with >10% missing reads, SNPs with minor allele frequency <5%, and loci with >50% heterozygosity were removed, resulting in 107,705 high- confidence SNPs.

### 4.8. GWAS, Candidate Gene Prediction, and Favourable-Allele Identification

GWAS was performed using the BLINK model in GAPIT v3 [92], selected for its superior statistical power and computational efficiency over conventional single-locus models such as GLM and MLM [93,94]. BLINK employs Bayesian Information Criterion within a fixed-effect framework and utilises linkage disequilibrium-based grouping, enabling more accurate detection of marker-trait associations [92,94]. Significant MTAs were identified using the Bonferroni threshold [95] and visualised using Manhattan and Q–Q plots in R (v4.4.1). Candidate genes were predicted by scanning ±150 kb around significant SNPs, consistent with reported linkage disequilibrium (LD) decay (100–300 kb) in rice [96,97]. Peak SNPs were labelled in the format qtrait.chromosome.nthSNP (e.g., qGP12.1). SNP positions were compared with previously reported QTLs and annotated genes in RAP-DB [98]. For each significant MTA, phenotypic means of the alleles were compared, and the allele associated with the higher mean performance was defined as the favourable allele. Where multiple MTAs were detected for a trait, the effects of different favourable-allele combinations were estimated [65]. Superior allelic combinations and the corresponding genotypes were visualised using an UpSet plot (UpSetR) [99], considering only genotypes possessing at least one favourable allelic combination.

### 4.9. Prediction Modelling and Stage-to-Stage Comparison

Prediction modelling used stratified sampling to divide the dataset into training and testing sets using the caret package in R, where stratification was based on salinity tolerance categories (HT, T, MT, S, and HS) derived from AMFVs to ensure proportional representation of each class. An alluvial plot (ggalluvial) was used to compare genotype tolerance across stages based on AMFV class. Accordingly, 80% of genotypes from each category were used for training and the remaining 20% for testing. Multicollinearity among predictors was assessed using the variance inflation factor (VIF) implemented in the car package in R [100]. Because predictors showed high multicollinearity, regularisation was applied. Alpha values (0–1) were evaluated using k-fold cross-validation, and the optimal alpha was selected using MSE, RMSE, NMSE, R^2^, and adjusted R^2^ using the glmnet package in R [101]. LASSO regression was fitted to germination-stage data and Elastic Net regression to early seedling-stage data. Unstandardised coefficients and intercepts were used to construct prediction equations. Model performance was evaluated in the testing set by comparing predicted salinity tolerance scores with observed AMFVs. Trait-level predictive strength was assessed using R^2^. 

## 5. Conclusions

**Figure 10:**
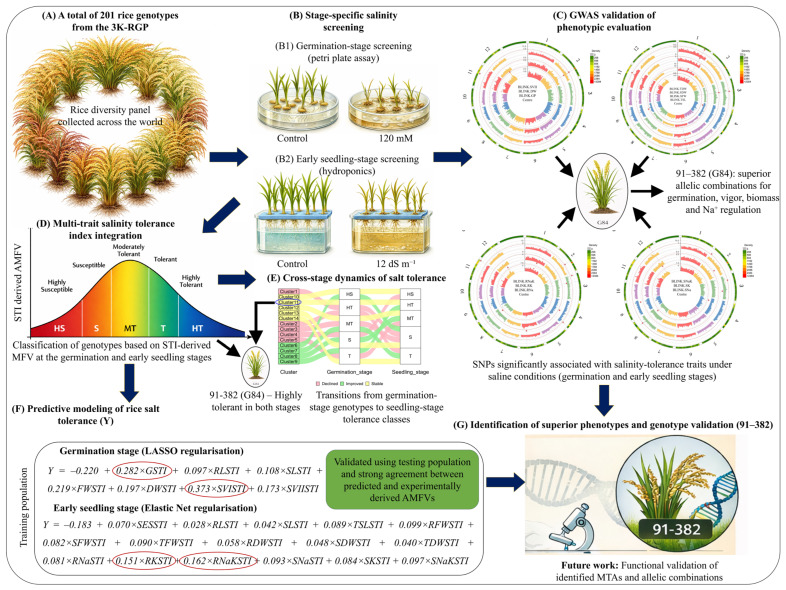
A total of 201 rice genotypes were evaluated for salinity tolerance using stage-specific phenotyping. (**A**) A globally diverse rice panel representing 37 countries was assembled. (**B**) Salinity screening was conducted at two developmental stages: (**B1**) the germination stage using a Petri plate assay under control and 120 mM NaCl, and (**B2**) the early seedling stage using a hydroponic system under control and 12 dS m^−1^ salinity. (**C**) Genome-wide association studies (GWAS) identified significant marker (SNP)-trait associations (MTAs) under saline conditions at both germination and early seedling stages and further resolved superior allelic combinations, particularly in genotype 91–382 (G84). Numerals correspond to chromosome numbers, and each color represents a different chromosome. (**D**) Multiple Stress Tolerance Indices (STIs) were integrated using the average membership function value (AMFV) to classify genotypes into five tolerance categories: highly susceptible (HS), susceptible (S), moderately tolerant (MT), tolerant (T), and highly tolerant (HT). (**E**) Cross-stage dynamics analysis tracked changes in genotype performance between tolerance classes from germination to early seedling stages, distinguishing stable, improved, and declined responses. (**F**) Predictive models of salinity tolerance were developed using regularisation approaches, with LASSO regularisation applied at the germination stage and Elastic Net regularisation at the early seedling stage, using 80% of the population for training and 20% for independent testing. The encircled coefficients indicate traits with higher contributions to the predictive model. (**G**) Integration of phenotypic evaluation, GWAS results, and prediction outputs identified genotype 91–382 as a superior line combining favourable alleles for germination, vigour, biomass, and ion homeostasis, providing a strong basis for future functional validation of key MTAs and allelic combinations. The rice plant illustrations in panels A, B, C, and G were generated using an artificial intelligence-based tool (ChatGPT, OpenAI, GPT-5.3 model).

Genotype 91-382 consistently outperformed other lines at both the germination and early seedling stages, demonstrating strong physiological resilience and effective ionic regulation under salinity. Integrating STI with MFV/AMFV strengthened genotype classification, and GWAS independently supported these phenotypes by identifying novel, functional, and gene-proximal SNP loci and favourable multilocus allele combinations associated with tolerance. Cross-validated prediction models showed that biomass- and vigour-related STIs primarily determined tolerance at germination, whereas ion-related STIs best predicted tolerance at the early seedling stage, enabling robust prediction of MFV-based tolerance scores across diverse genotypes. Together, physiological indices, genomic evidence, and predictive modelling provide a scalable framework to identify elite donors and accelerate salt-tolerant rice breeding, with future work (Figure 10) focusing on functional validation of the identified MTAs, development of diagnostic markers, and their deployment in marker-assisted and haplotype-based breeding using favourable allelic combinations.

## Figures and Tables

**Table 1:** AMFV-based classification thresholds for salinity tolerance.

Category	Standard Formula	Germination ($\bar{X}$ = 0.44, SD = 0.19)	Early Seedling ($\bar{X}$ = 0.43, SD = 0.18)
HT	AMFV ≥ $\bar{X}+1.64$SD	AMFV ≥ 0.74	AMFV ≥ 0.72
T	$\bar{X}+1$SD ≤ AMFV < $\bar{X}+1.64$SD	0.62 ≤ AMFV < 0.74	0.61 ≤ AMFV < 0.72
MT	$\bar{X}-1$SD ≤ AMFV < $\bar{X}$+ 1SD	0.25 ≤ AMFV < 0.62	0.26 ≤ AMFV < 0.61
S	$\bar{X}$ − 1.64SD ≤ AMFV < $\bar{X}$ − 1SD	0.14 ≤ AMFV < 0.25	0.14 ≤ AMFV < 0.26
HS	AMFV < $\bar{X}$ − 1.64SD	AMFV < 0.13	AMFV < 0.14

AMFV—average membership function value, $\bar{X}$—mean, SD—standard deviation, HT—highly tolerant, T—tolerant, MT—moderately tolerant, S—susceptible, HS—highly susceptible.

**Table 2:** Representative MTAs and their nearest candidate genes detected around significant SNPs.

MTA	Ch	Position of SNP	FA	AA	Candidate Gene (Nearest)	Distance (kb)
qGP12.1	12	21124440	T	A	Novel	–
qGP12.2	12	21124449	G	A	Novel	–
qSFW1.1	1	24001919	T	C	Novel	–
qRNa12.1	12	21124440	T	A	Novel	–
qSNa12.1	12	21124440	T	A	Novel	–
qSNaK12.1	12	21124440	T	A	Novel	–
qSNaK2.1 *	2	839096	G	A	Os02g0115900 (*OsPHD9*)	0.353
qRNaK4.1 *	4	33419501	A	G	Os04g0655300 (*OsRLCK168*)	0.864
qRNa8.1 *	8	20409355	C	T	Os08g0425100 (*OsDRP2C*)	1.13
qRK3.1	3	10391265	T	C	Os03g0297100 (*OsRPS7a*)	2.39
qRNaK3.1	3	510532	A	G	Os03g0108600 (*OsBIRH1*)	4.61
qSFW9.1	9	10735723	T	C	Os09g0344500 (*ASMT1*)	4.62
qSNa8.1	8	20387112	G	A	Os08g0424500 (*OsBADH2*)	7.28
qRNaK2.1	2	21423766	C	T	Os02g0564100 (*OsPHD9*)	9.88
qRK4.1	4	19863474	A	G	Os04g0401700 (*OsHAK1*)	23.2
qTSL4.1, qSNa4.1	4	5289668	C	T	Os04g0178300 (*OsCPS4*)	28.39
qSNaK3.1 **	3	7209498	A	G	Os03g0236200 (*OsGAD3*)	28.42
qRK6.1	6	22309915	G	A	Os06g0575400 (*OsSDRLK5*)	42.17
qSDW1.1, qTDW1.1	1	35456843	T	C	Os01g0829800 (*MDH1*)	42.18
qSDW6.1, qTDW6.1	6	12287211	C	A	Os06g0318500 (*OsNHX4*)	46.02
qSDW3.1, qTDW3.1	3	16109980	A	G	Os03g0397400 (*OsNCX7*)	48.96

MTA—marker–trait association; Ch—chromosome; FA—favourable allele; AA—alternate allele; * indicates the SNP is located within the candidate gene; ** indicates a non-synonymous SNP. Abbreviations are defined in the List of Abbreviations.

**Table 3:** Homozygous allelic combinations showing the highest and lowest mean trait values among loci with multiple significant SNPs.

Trait	Associated MTAs	Allelic Combination with Highest Mean	Mean Value	Allelic Combination with Lowest Mean	Mean Value
GP	qGP12.1, qGP12.2	TG	83.8	AA	67.17
SVI II	qSVII2.1, qSVII11.1	TT	1.19	CC	0.49
SFW (g)	qSFW1.1, qSFW9.1, qSFW11.1	TTG	0.986	CCG	0.173
SDW (g)	qSDW1.1, qSDW3.1, qSDW6.1	CAC	0.097	CGA	0.018
TDW (g)	qTDW1.1, qTDW3.1, qTDW6.1, qTDW11.1	CACT	0.103	CGAC	0.02
RNa (mmol g^−1^ DW)	qRNa1.1, qRNa8.1, qRNa12.1	TTA	12.06	CCT	1.75
RK (mmol g^−1^ DW)	qRK3.1, qRK4.1, qRK6.1	TAG	2.57	TGA	0.25
RNaK	qRNaK2.1, qRNaK3.1, qRNaK4.1, qRNaK11.1	TGGC	36.1	CAGT	3.49
SNa (mmol g^−1^ DW)	qSNa4.1, qSNa8.1, qSNa12.1	TAA	10.47	CGT	1.98
SK (mmol g^−1^ DW)	qSK2.1, qSK3.1, qSK9.1	CAA	3.24	CGT	0.82
SNaK	qSNaK2.1, qSNaK3.1, qSNaK8.1, qSNaK12.1	AGTT	16	GACT	1.47

A—adenine; C—cytosine; G—guanine; T—thymine.

## Data Availability

The data presented in this study are available in the article and Supplementary Materials. Additional datasets, including phenotypic data and large genotypic files, are available from the corresponding author on reasonable request. The SNP genotypic data used in this study were obtained from the Rice SNP-Seek Database.

## Supplementary Materials

The supporting information can be downloaded at: https://www.mdpi.com/article/10.3390/plants15071046/s1, Figure S1: Quantile–quantile (Q–Q) plot showing the distribution of observed and expected −log10(*p*) values obtained from the BLINK model; Figure S2: Co-association of significant marker–trait associations across chromosomes; Table S1: Details of the rice genotypes used in the current study; Table S2: Classification of genotypes based on AMFV derived from STI under salinity stress (germination stage); Table S3: Classification of genotypes based on AMFV derived from STI under salinity stress (early seedling stage); Table S4: Details of cross-stage dynamics of salt tolerance in rice genotypes; Table S5: List of SNP markers and their allelic effects identified for salinity tolerance traits in rice through GWAS at germination and early seedling stage; Table S6: List of all available homozygous allelic combinations of MTAs; Table S7: Predictive modelling of rice salt tolerance using LASSO regularization-details of metrics (germination stage); Table S8: Predictive modelling of rice salt tolerance using LASSO regularization-details of metrics (early seedling stage).

## Abbreviations

The following abbreviations are used in this manuscript:

3K-RGP	3000 Rice Genome Project
3K-RDP	3000 Rice Diversity Panel
AMFV	Average Membership Function Value
CRD	Completely Randomized Design
DW	Dry Weight
DWSTI	Dry Weight Stress Tolerance Index
EC	Electrical Conductivity
FW	Fresh Weight
FWSTI	Fresh Weight Stress Tolerance Index
GP	Germination Percentage
GSTI	Germination Stress Tolerance Index
GWAS	Genome-Wide Association Study
HT	Highly Tolerant
HS	Highly Susceptible
IRGC	International Rice Genebank Collection
IRRI	International Rice Research Institute
K^+^	Potassium ion
LD	Linkage Disequilibrium
LASSO	Least Absolute Shrinkage and Selection Operator
MFV	Membership Function Value
MLM	Mixed Linear Model
MSE	Mean Squared Error
MT	Moderately Tolerant
MTA	Marker–Trait Association
Na^+^	Sodium ion
NMSE	Normalized Mean Squared Error
PVE	Phenotypic Variance Explained
R^2^	Coefficient of Determination
RDW	Root Dry Weight
RDWSTI	Root Dry Weight Stress Tolerance Index
RFW	Root Fresh Weight
RFWSTI	Root Fresh Weight Stress Tolerance Index
RK	Root Potassium Content
RKSTI	Root Potassium Stress Tolerance Index
RL	Root Length
RLSTI	Root Length Stress Tolerance Index
RNa	Root Sodium Content
RNaK	Root Sodium/Potassium Ratio
RNaKSTI	Root Sodium/Potassium Stress Tolerance Index
RNaSTI	Root Sodium Stress Tolerance Index
RMSE	Root Mean Squared Error
SD	Standard Deviation
SDW	Shoot Dry Weight
SDWSTI	Shoot Dry Weight Stress Tolerance Index
SE	Standard Error
SES	Standard Evaluation Score
SESSTI	Standard Evaluation Score Stress Tolerance Index
SFW	Shoot Fresh Weight
SFWSTI	Shoot Fresh Weight Stress Tolerance Index
SK	Shoot Potassium Content
SKSTI	Shoot Potassium Stress Tolerance Index
SL	Shoot Length
SLSTI	Shoot Length Stress Tolerance Index
SNa	Shoot Sodium Content
SNaK	Shoot Sodium/Potassium Ratio
SNaKSTI	Shoot Sodium/Potassium Stress Tolerance Index
SNaSTI	Shoot Sodium Stress Tolerance Index
SNP	Single Nucleotide Polymorphism
STI	Stress Tolerance Index
SVI	Seedling Vigour Index I
SVII	Seedling Vigour Index II
SVISTI	Seedling Vigour Index I Stress Tolerance Index
SVIISTI	Seedling Vigour Index II Stress Tolerance Index
T	Tolerant
TDW	Total Dry Weight
TDWSTI	Total Dry Weight Stress Tolerance Index
TFW	Total Fresh Weight
TFWSTI	Total Fresh Weight Stress Tolerance Index
TSL	Total Seedling Length
TSLSTI	Total Seedling Length Stress Tolerance Index
VIF	Variance Inflation Factor
