# Supplementary material for: GWAS and Regularised Regression Identify SNPs Associated with Candidate Genes for Stage-Specific Salinity Tolerance in Rice
Source: Plants (Basel). 2026 Mar 28;15(7):1046. doi: 10.3390/plants15071046 (PMC13075185; doi:10.3390/plants15071046)
Supplement: Supplementary file 1 [file plants-15-01046-s001.zip › plants-4141471-supplementary/Supplementay file_2_27.3.26.pdf]

Supplementary Table S7. Predictive Modelling of Rice Salt Tolerance Using Lasso Regularization - details of metrics (Germination stage)

Alpha	MSE	RMSE	NMSE	R2	Adjusted_R2	λ_Min	λ_1SE
0	3.45595E-05	0.0059	0.0010	0.9990	0.9990	0.0255	0.0255
0.1	3.22461E-05	0.0057	0.0009	0.9991	0.9990	0.0153	0.0168
0.2	3.28481E-05	0.0057	0.0009	0.9991	0.9990	0.0111	0.0122
0.3	3.47971E-05	0.0059	0.0010	0.9990	0.9990	0.0089	0.0098
0.4	3.20719E-05	0.0057	0.0009	0.9991	0.9990	0.0067	0.0073
0.5	3.44053E-05	0.0059	0.0010	0.9990	0.9990	0.0059	0.0064
0.6	3.38185E-05	0.0058	0.0010	0.9990	0.9990	0.0049	0.0054
0.7	3.33707E-05	0.0058	0.0009	0.9991	0.9990	0.0042	0.0046
0.8	3.34279E-05	0.0058	0.0009	0.9991	0.9990	0.0037	0.0040
0.9	3.43032E-05	0.0059	0.0010	0.9990	0.9990	0.0033	0.0036
1*	3.19707E-05	0.0057	0.0009	0.9991	0.9991	0.0027	0.0032

*Alpha value of 1 has the lowest MSE and highest R^2; MSE, Mean Squared Error; RMSE, Root Mean Squared Error; NMSE, Normalized Mean Squared Error; R^2, Coefficient of Determination; Adjusted R^2, Adjusted Coefficient of Determination; λ_Min, regularization parameter value that gives the minimum cross-validated error; λ_1SE, regularization parameter value within one standard error of the minimum cross-validated error.

Supplementary Table S8. Predictive Modelling of Rice Salt Tolerance Using Lasso Regularization - details of metrics (Early seedling stage)

Alpha	MSE	RMSE	NMSE	R2	Adjusted_R2	λ_Min	λ_1SE
0	2.98E-05	0.00546	0.0010	0.9990	0.9989	0.0335	0.0368
0.1*	2.81E-05	0.00530	0.0009	0.9991	0.9990	0.0167	0.0183
0.2	2.91E-05	0.00539	0.0009	0.9991	0.9989	0.0110	0.0121
0.3	3.02E-05	0.00549	0.0010	0.9990	0.9989	0.0081	0.0089
0.4	2.97E-05	0.00545	0.0010	0.9990	0.9989	0.0061	0.0066
0.5	2.91E-05	0.00539	0.0009	0.9991	0.9990	0.0048	0.0053
0.6	2.91E-05	0.00539	0.0009	0.9991	0.9990	0.0040	0.0044
0.7	2.96E-05	0.00544	0.0010	0.9990	0.9989	0.0035	0.0038
0.8	3.05E-05	0.00553	0.0010	0.9990	0.9989	0.0030	0.0033
0.9	2.90E-05	0.00538	0.0009	0.9991	0.9990	0.0025	0.0030
1	3.04E-05	0.00551	0.0010	0.9990	0.9989	0.0022	0.0022

*Alpha value of 0.1 has the lowest MSE and highest R^2; MSE, Mean Squared Error; RMSE, Root Mean Squared Error; NMSE, Normalized Mean Squared Error; R^2, Coefficient of Determination; Adjusted R^2, Adjusted Coefficient of Determination; λ_Min, regularization parameter value that gives the minimum cross-validated error; λ_1SE, regularization parameter value within one standard error of the minimum cross-validated error.

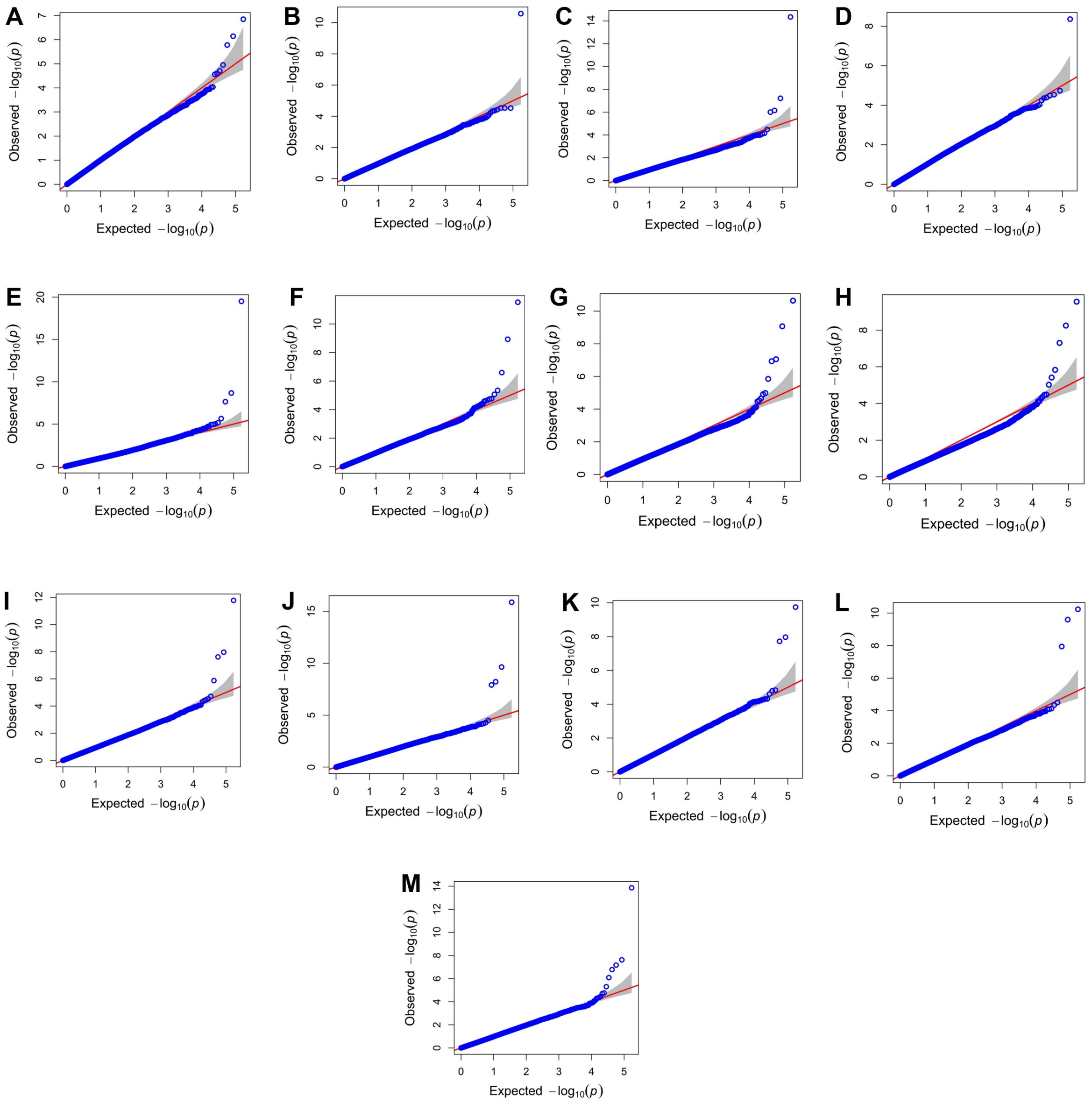

Supplementary Figure S1. Quantile–quantile (Q–Q) plot showing the distribution of observed and expected −log10(p) values obtained from the BLINK model: A. Germination percentage, B. Dry weight, C. Seedling vigor index II (Germination stage), D. Total Seedling Length, E. Shoot fresh weight, F. Shoot dry weight, G. Total dry weight, H. Root Na^+, I. Root K^+, J. Root Na^+/K^+, K. Shoot Na^+, shoot K^+, and shoot Na^+/K^+ ratio (Early seedling stage)

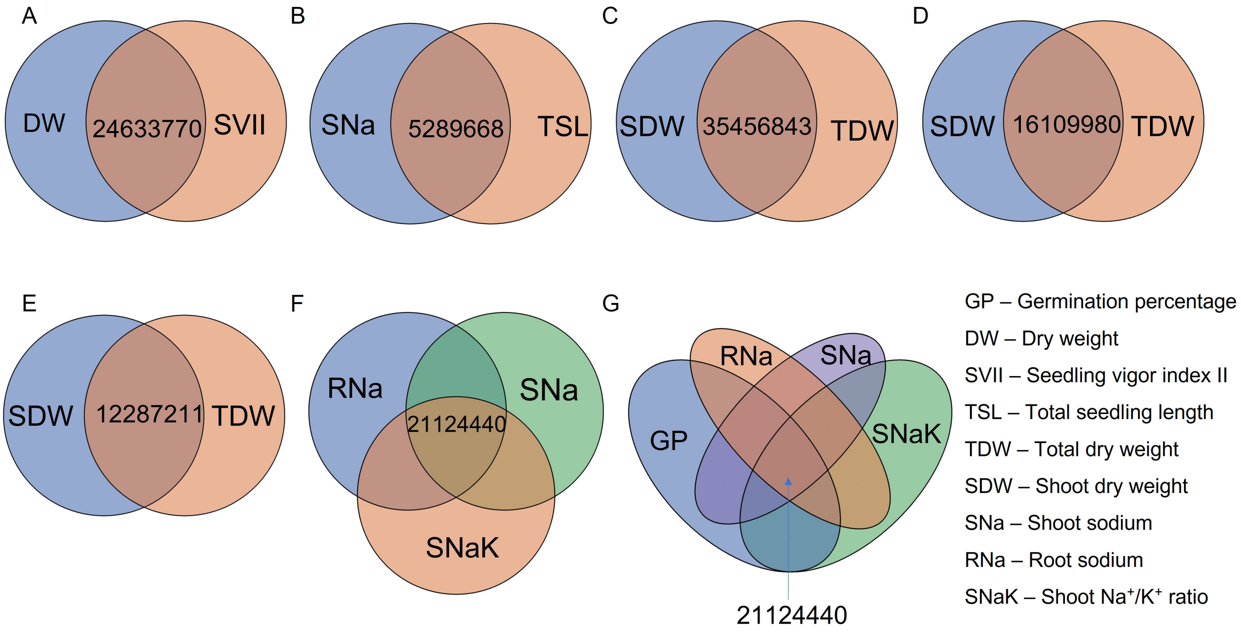

Supplementary Figure S2. Co-association of significant marker–trait associations across chromosomes: A. Dry weight and seedling vigor index II (germination stage, Chr 1); B. Shoot sodium and Total seedling length (early seedling stage, Chr 4); C. Shoot dry weight and Total dry weight (early seedling stage, Chr 2); D. Shoot dry weight and Total dry weight (early seedling stage, Chr 3); E. Shoot dry weight and Total dry weight (early seedling stage, Chr 6); F. Root sodium, shoot sodium, and shoot Na^{+}/K^{+} (early seedling stage, Chr 12); G. Germination percentage, root sodium, shoot sodium, and shoot Na^{+}/K^{+} (germination and early seedling stages, Chr 12). Numbers indicate genomic positions of common SNPs; Ch denotes chromosome
